# Evaluation of *Annona muricata* Acetogenins as Potential Anti-SARS-CoV-2 Agents Through Computational Approaches

**DOI:** 10.3389/fchem.2020.624716

**Published:** 2021-01-27

**Authors:** Shashanka K. Prasad, Sushma Pradeep, Chandan Shimavallu, Shiva Prasad Kollur, Asad Syed, Najat Marraiki, Chukwuebuka Egbuna, Mihnea-Alexandru Gaman, Olga Kosakowska, William C. Cho, Kingsley Chukwuemeka Patrick-Iwuanyanwu, Joaquín Ortega Castro, Juan Frau, Norma Flores-Holguín, Daniel Glossman-Mitnik

**Affiliations:** ^1^ Department of Biotechnology and Bioinformatics, School of Life Sciences, JSS Academy of Higher Education and Research, Mysuru, India; ^2^ Department of Sciences, Amrita School of Arts and Sciences, Amrita Vishwa Vidyapeetham, Mysuru, India; ^3^ Department of Botany and Microbiology, College of Science, King Saud University, Riyadh, Saudi Arabia; ^4^ Department of Biochemistry, Faculty of Natural Sciences, Chukwuemeka Odumegwu Ojukwu University, Anambra State, Nigeria; ^5^ Department of Biochemistry, University of Port Harcourt, Choba, Nigeria; ^6^ Faculty of Medicine, Carol Davila University of Medicine and Pharmacy, Bucharest Romania; ^7^ Center of Hematology and Bone Marrow Transplantation, Fundeni Clinical Institute, Bucharest, Romania; ^8^ Center of Hematology and Bone Marrow Transplantation, Fundeni Clinical Institute, Bucharest, Romania; ^9^ Department of Vegetable and Medicinal Plants, Institute of Horticulture Sciences, Warsaw University of Life Sciences - SGGW, Warsaw, Poland; ^10^ Department of Clinical Oncology, Queen Elizabeth Hospital, Kowloon, Hong Kong SAR, China; ^11^ Departament de Química, Universitat de les Illes Balears, Palma de Malllorca, Spain; ^12^ Laboratorio Virtual NANOCOSMOS, Departamento de Medio Ambiente y Energía, Centro de Investigación en Materiales Avanzados, Chihuahua, Mexico; ^13^ World Bank Africa Centre of Excellence for Public Health and Toxicological Research (ACE-PUTOR), University of Port Harcourt, Nigeria

**Keywords:** Annonaceous acetogenins, COVID-19, *in silico*, molecular docking, molecular dynamics simulation study, *Annona muricata*

## Abstract

*Annona muricata*, a tropical plant which has been extensively used in ethnomedicine to treat a wide range of diseases, from malaria to cancer. Interestingly, this plant has been reported to demonstrate significant antiviral properties against the human immunodeficiency virus, herpes simplex virus, human papilloma virus, hepatitis C virus and dengue virus. Additionally, the bioactive compounds responsible for antiviral efficacy have also shown to be selectively cytotoxic while inhibiting tumorigenic cell growth without affecting the normal cell growth. Annonaceous Acetogenins are a class of bioactive compounds exclusive to the Annonaceae family at which the plant *A. muricata* belongs. In the current study, we have created a library of Acetogenins unique to the plant, comprising of Annomuricin A, Annomuricin B, Annomuricin C, Muricatocin C, Muricatacin, *cis*-Annonacin, Annonacin-10-one, *cis*-Goniothalamicin, Arianacin and Javoricin, for *in silico* and theoretical evaluations against the SARS-CoV-2 spike protein in an attempt toward promotion of plant based drug development for the current pandemic of coronavirus disease 2019 (COVID-19). We found that all the Acetogenins showing *in silico* spike protein significantly docking with good binding affinities. Moreover, we envision *A. muricata* Acetogenins can be further studied by *in vitro* and *in vivo* models to identify potential anti-SARS-CoV-2 agents.

## 1 Introduction

*Annona muricata* (L.), commonly called Soursop (English) or Lakshmanaphala (Kannada), is a tropical plant known for its wide range of applications in ethnomedicinal practices (Moghadamtousi et al., 2015; Gavamukulya et al., 2017; Prasad et al., 2019). Belonging to the Annonaceae family, *A. muricata* has grabbed the attention of global scientific communities toward investigating the medicinal significance of its constituent phytochemicals. Found exclusive to the Annonaceae family, the annonaceous Acetogenins are one class of phytochemicals that have been investigated thoroughly for their potent biomedical properties. Chemically, Acetogenins are the metabolic derivatives of long-chain fatty acids derived via the polyketide pathway (Prasad et al., 2019). Over 500 Acetogenins have been reported across the family (Moghadamtousi et al., 2015; Liaw et al., 2016). Acetogenins have been reported to show significant antiviral activities against herpes simplex virus - I (HSV-I) (Padma et al., 1998), herpes simplex virus - II (HSV-II) (Betancur-Galvis et al., 1999), human papillomavirus (HPV) (Donne et al., 2017), hepatitis C virus (HCV) (Apriyanto et al., 2018), dengue virus type 2 (DENV-2) (Wahab et al., 2018), human immunodeficiency virus - I (HIV-I) (van de Venter et al., 2014; Gavamukulya et al., 2017). The above observations confirm the broad-spectrum antiviral activity of *A. muricata* phytochemicals, which may also be responsible for the other biomedical properties. In this first combination of theoretical and computational studies on annonaceous Acetogenins, we have created a library of Annomuricin A, Annomuricin B, Annomuricin C, Muricatocin C, Muricatacin, *cis*-Annonacin, Annonacin-10-one, *cis*-Goniothalamicin, Arianacin and Javoricin, to investigate their *in silico* anti-SARS-CoV-2 activity. The above compounds are unique to *A. muricata* and are the only reported Acetogenins with potent anti-breast cancer activity. This study provides evidence for drug development exercises toward mitigation of the current pandemic, coronavirus disease 2019 (COVID-19) caused by the SARS-CoV-2 virus.

Chemoinformatics is a collection of different procedures for the study and organization of chemical information, it is a useful approach for the development of new medical drugs in the pharmaceutical industry. Chemoinformatics applications are valuable for the prediction of the molecular properties of many systems based on the knowledge of previously studied molecular structures and computational displaying of the same by considering the close relationship between biological data and organic structures and energies. This is the way that molecular descriptors can be related with molecular properties (Guha and Bender, 2012).

There are numerous *in silico* studies pertaining to the understanding of standard drug(s)/phytochemical(s) binding to SARS-CoV-2 surface proteins, most of which are considered a potential drug targets (Boopathi et al., 2020). Gupta et al. reported similar ADME properties apart from the ‘Lipsinki Rule of 5′ for phytochemicals Belachinal, Macaflavanone E and Vibsanol B, all of which interacted mainly with the VAL25 and PHE26 aminThuso acids to facilitate the binding to SARS-CoV-2 E protein Gupta et al. (2020). In an alternative approach, Sarma et al. suggested the targeting of coronavirus nucleocapsid (N) protein RNA-binding N-terminal domain (NTD). In this study, Two suitable binders, one theophylline derivative and one 3,4-dihydropyrimidone class molecule, were identified to target the 2OFZ, an ultrahigh resolution crystal structure of RNA binding domain of CoV N protein of resolution 1.1 Å in monoclinic form Sarma et al. (2020). Meanwhile, another study focused on understanding the interaction of FDA approved drugs such as remdesivir, saquinavir and duranavir, as potent chymotrypsin-like protease (3CL^pro^) of the SARS-CoV-2 virus in an attempt to provide for drug repurposing and expediting the drug discovery process (Khan et al., 2020b). Similarly, 3CL^pro^ as well as 2′-O-ribosemethyltransferase (2′-O-MTase) were reported druggable targets by Khan et al. for systematic drug repurposing of paritaprevir and raltegravir for 3CL^pro^ and dolutegravir and bictegravir for 2′-O-MTase (Khan et al., 2020a). The SARS-CoV-2 spike glycoprotein has been extensively reviewed as a potential target for COVID-19 therapeutics (Basit et al., 2020). The above findings laid the foundation for the current study. Other studies on the efficacy of phytochemicals against SARS-CoV-2 have been conducted for instance, Gupta et al. (2020) have analyzed the SARS-CoV-2 envelope (E) protein binding and inhibition efficiency of the plant-derived Belachinal, Macaflavanone E and Vibsanol B Gupta et al. (2020).

## 2 Materials and Methods

### 2.1 Library and Macromolecule Preparation

Coronaviruses (CoVs) are single-stranded RNA viruses that infect a wide range of hosts to cause pulmonary complications which may range from mild to severe or fatal. The surface-exposed spike glycoprotein, or S protein, plays a major role in infecting the host cell membranes to release viral RNA molecules. Thereby making it a target of choice for COVID-19 therapeutics, where the vaccines or any small agents designed to cure or control the disease must primarily target and block or inhibit the activity of these spike proteins. In this study, ten Acetogenins unique to *A. muricata* have been evaluated for the SARS-CoV-2 inhibition potential, by means of targeting the spike protein. The *in silico* molecular docking results of ten Acetogenins against the SARS-CoV-2 spike protein were compared with the molecular docking results of dexamethasone.

In this regard, the crystal structure of SARS-CoV-2 spike receptor-binding domain bound with angiotensin converting enzyme 2 (ACE2) (PDB ID- 6M0J) (Supplementary Figure S1) was considered and downloaded from the biological structural database of proteins, i.e. Protein Data Bank (PDB). The 3-dimensional structure of the protein was checked for the presence of any pre-existing ligands and the water molecules were deleted or removed and the resultant protein structure was saved in .pdb format for the further validation.

The protein validation was carried out using RAMPAGE (http://mordred.bioc.cam.ac.uk/~rapper/rampage.php) that gives the Ramachandran plot analysis of the protein predicting its amino acid residue details in both the allowed and the favored regions. The protein was found to have 94.6% number of residues in the favored region, with 1.8% of residues in the allowed region and thus was presumed suitable for docking.

The binding or the active site pocket residues where the ligands interact with the protein to inhibit its action were selected based on the literature survey and the grid box was generated around the binding pocket residues present in between the coronavirus spike protein and the ACE2 receptor.

### 2.2 Ligand Optimization

The 3D structural files of the ten Acetogenin ligand molecules were required to carry out the SARS-CoV-2 spike protein docking interaction. For which, an initial 2D structure was sketched and geometrically cleaned using the ChemSketch freeware software by the ACD labs, Canada. The final 2D structure was saved as chemical markup language files to be further converted to 3D structure files using the OpenBabel chemical toolbox, an open-collaborative project, by generating the 3D coordinates upon addition of hydrogen atoms into the structure. Finally, the resultant pdb files were analyzed for their structure, followed by the second geometrical clean-up to add up any leftover hydrogens and resaved in the. pdb format using the ArgusLab software (A molecular modeling, graphics and drug design program) before further interaction analysis.

To perform the comparative docking studies, the .sdf format file of the approved drug for COVID-19, dexamethasone (https://pubchem.ncbi.nlm.nih.gov/compound/Dexamethasone; PubChem CID: 5743), was downloaded from PubChem, a free online database of chemical molecules (https://pubchem.ncbi.nlm.nih.gov/). Similarly, the chemical structures, in. sdf file format, of Annomuricin A (https://pubchem.ncbi.nlm.nih.gov/compound/Annomuricin-A; PubChem CID: 157682), Annomuricin B (https://pubchem.ncbi.nlm.nih.gov/compound/Annomuricin-B; PubChem CID: 44575650), Annomuricin C (https://pubchem.ncbi.nlm.nih.gov/compound/Annomuricin-C; PubChem CID: 11758463), Muricatocin C (https://pubchem.ncbi.nlm.nih.gov/compound/Muricatocin-C; PubChem CID: 44584147), Muricatacin (https://pubchem.ncbi.nlm.nih.gov/compound/Muricatacin; PubChem CID: 3035657), *cis*-Annonacin (https://pubchem.ncbi.nlm.nih.gov/compound/cis-Annonacin; PubChem CID: 10698767), Annonacin-10-one (https://pubchem.ncbi.nlm.nih.gov/compound/Annonacin-10-one; PubChem CID: 180161), *cis*-Goniothalamicin (https://pubchem.ncbi.nlm.nih.gov/compound/cis-Goniothalamicin; PubChem CID: 10722235), Arianacin (https://pubchem.ncbi.nlm.nih.gov/compound/Arianacin; PubChem CID: 10698768) and Javoricin (https://pubchem.ncbi.nlm.nih.gov/compound/Javoricin; PubChem CID: 10326193), were obtained from PubChem. The downloaded files were converted to .pdb file by following the same steps as mentioned in the ligand preparation using the OpenBabel and ArgusLab software.

### 2.3 Molecular Docking and Interaction Studies

The molecular docking studies for the prepared ligands structural files were carried out to evaluate their inhibition potential, against the prepared and validated SARS-CoV-2 spike protein, using AutoDock Vina in PyRx an open-source virtual screening tool (https://pyrx.sourceforge.io/). Initially, the above obtained 3D structural files (.pdb) of the protein were uploaded alongside the ligand file (.pdb) of choice. The loaded .pdb files were generated into .pdbqt files comprising of whole protein molecule that was prepared and validated earlier with added hydrogen and charges along with the ligands in .pdbqt files. At this juncture, the binding site residues were selected and a grid box assigned to surround them in a manner that all the binding site residues were within the grid box. Finally, the docking process was run using the generic algorithm parameter.

The docking results were obtained in the form of conformations or poses, with eight different docking poses for every ligand, with the binding affinity values and their RMSD values against their respective protein or macromolecule. The best docked pose was saved in the .pdb format to check for their interaction against the protein.

### 2.4 Molecular Dynamics Simulation Studies

The ligand *cis*-Annonacin- SARS-CoV-2 spike protein complex that has obtained the highest binding affinity and has interacted very well was selected for the molecular dynamics (MD) simulation studies. The MD simulation was carried out using GROMACS, forced with CHARMM27 all-atom force field. The parameter files for the ligand required to run the simulation were generated using SwissParam online server. TIP3P water model was used to solvate the system and counter ions were added to neutralize the system. The steepest descent algorithm was used to minimize the structures and the system was equilibrated by applying position restrains by performing simulations using NVT ensembles followed by NPT. The equilibration was run for 20 picoseconds (ps) at 300 K temperature and 1 bar pressure. After equilibrating the system, the MD run was carried out for the system at 30 ns. The energies and coordinates were saved at every 10 ps for analysis purpose.

### 2.5 Computational Details

The determination of the conformers of the molecules considered in this study was performed by resorting to MarvinView 17.15 available from ChemAxon (Budapest, Hungary) by doing Molecular Mechanics calculations through the overall MMFF94 force field (Halgren, 1996a; Halgren, 1996b; Halgren, 1996c; Halgren and Nachbar, 1996; Halgren, 1999). This was followed by a geometry reoptimization by means of the Density Functional Tight Binding (DFTB) methodology (Frisch et al., 2016). The electronic properties of the studied molecules involved the use of MN12SX/Def2TZVP/H2O model chemistry on the previously optimized molecular structures because it has been shown that it allows the verification of the ‘Koopmans in DFT’ (KID) procedure (Flores-Holguín et al., 2019a; Flores-Holguín et al., 2019b) with the aid of the Gaussian09 software (Frisch et al., 2016) and the SMD solvation model (Marenich et al., 2009).

## 3 Results and Discussion

### 3.1 Molecular Docking Studies

To analyze the results, the best docked pose of the ligand and the protein were opened using PyMol and the obtained merged protein-ligand structures were saved in. pdb file to obtain a protein-ligand complex. The same thing was carried out for all the ten ligands including the standard Dexamethasone drug. The formation of bonded and non-bonded interactions of the ligands with the respective binding site amino acids of the protein were obtained using an online tool Protein-Ligand Interaction Profiler (https://projects.biotec.tu-dresden.de/plip-web/plip/index). The obtained PyMol session file was saved and the resultant interaction (hydrophobic interaction and hydrogen bonds) table was also saved, the dashed lines indicate hydrophobic interactions while the blue lines indicate hydrogen bonds formed between the amino acid residues of the protein with the ligands.

### 3.2 Ligands - Spike Protein Interaction

To explore the possible binding patterns of all the ligands selected for the study and interactions of the selected phytocompounds, Molecular Docking approaches were employed. The ten ligands along with the drug used for PyRx docking were evaluated on the basis of the obtained binding affinity results and their values interpreted on the basis of the strength which the ligand has bound to the macromolecule. The results were analyzed based on their interaction in the active site of the selected viral proteins. All the ten ligands were seen as interacting very well with the spike protein by forming binding affinity energies within the range of −5.3 to −7.7 kcal/mol whereas the drug molecule dexamethasone exhibited a binding affinity of −7.5 kcal/mol against the spike protein. The highest affinity of −7.7 kcal/mol interaction toward the protein was shown by *cis*-Annonacin which is better than the reference drug, while the lowest binding affinity was shown by Muricatacin. The compound *cis*-Annonacin showed the highest binding affinity of −7.7 kcal/mol with the binding site of the protein sharing five hydrogen bonds with PHE-390A, ARG-393A, GLN-409E, GLY-496E, TYR-505E and two each with ARG-403E and TYR-453E along with more hydrophobic interactions with ASN-33A, GLU-37A, PRO-389, ARG-403E, GLU-406E, LYS-417E, TYR-495E, PHE-497E and 2 with TYR-505E, while the lowest binding energy was shown by Muricatacin. The overall interaction of *cis*-Annonacin was shown to be comparatively better than the reference drug. The bond details of each ligand with the spike protein are shown in Table 1 (Supplementary Figures S2–S12).

**TABLE 1:** Binding affinity values of the docked ligand and number of hydrogen bonds formed.

Compound	Binding affinity values (Kcal/mol)	Number of hydrogen bonds
Dexamethasone	−7.5	6
Annomuricin A	−6.8	7
Annomuricin B	−6.7	4
Annomuricin C	−5.9	9
Muricatocin C	−6.4	7
Muricatacin	−5.3	3
*cis*-Annonacin	−7.7	9
Annonacin-10-one	−6.5	6
*cis*-Goniothalamicin	−6.3	6
Arianacin	−6.7	3
Javoricin	−6.3	7

### 3.3 Molecular Dynamics Simulation Study

MD simulation study was performed for the protein-ligand complex to explain their dynamic behavior. The spike protein-*cis*-Annonacin ligand interaction having minimum binding energy (strong binding affinity) was considered for further evaluation using molecular dynamics simulation to check their stability in complexes under simulated conditions. The simulation study provides the analysis of root mean square deviation (RMSD), radius of gyration (Rg), solvent accessible surface area (SASA), number of hydrogen bonds maintained throughout the simulation time and variation of secondary structure pattern between the protein and their complexes. The simulation was performed at the time duration of 10 ns with the native protein alone and in complex with ligand Annonacin.

**FIGURE 1:**
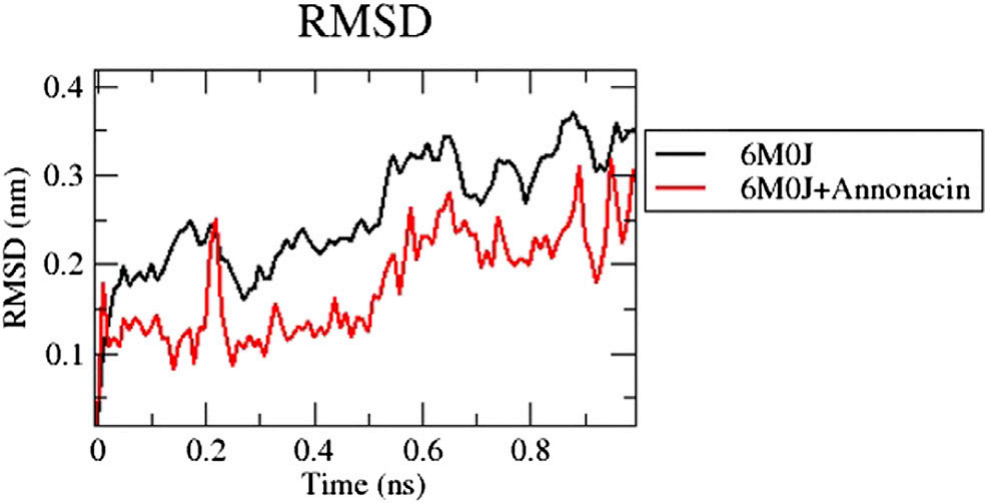
Analysis of RMSD of 6M0J alone and complexed with the ligand over 10 ns simulation run.

The RMSD plot in Figure 1 shows that the protein-ligand complex reached equilibrium approximately at 0.3–0.4 ns time and remaining showed stable trajectory with minimal deviation in 0.1–0.15 nm RMSD range, the protein structural flexibility are being reserved while it is in free form in complex with ligand. After the initial fluctuations the ligand bound to the protein reached equilibrium.

**FIGURE 2:**
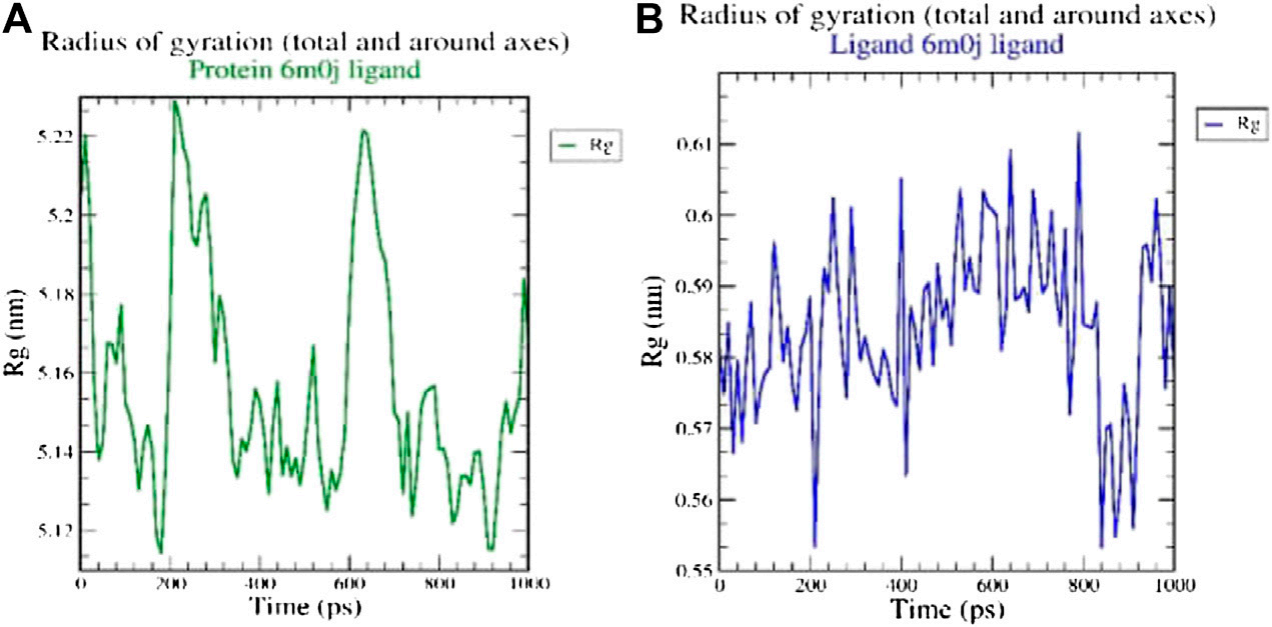
Analysis of radius of gyration (Rg) of **(A)** 6M0J alone and **(B)** complex with the ligand over 10 ns simulation run.

The radius of gyration (Rg) considers the varied masses calculated to root mean square distances considering the central axis of rotation. The Rg plot (Figure 2 considers the capability, shape and folding during the each time step of the whole trajectory throughout the simulation. The protein and their respective ligand complex exhibited the similar pattern of Rg value with the deviation in the range of 0.55–5.22 nm.

**FIGURE 3:**
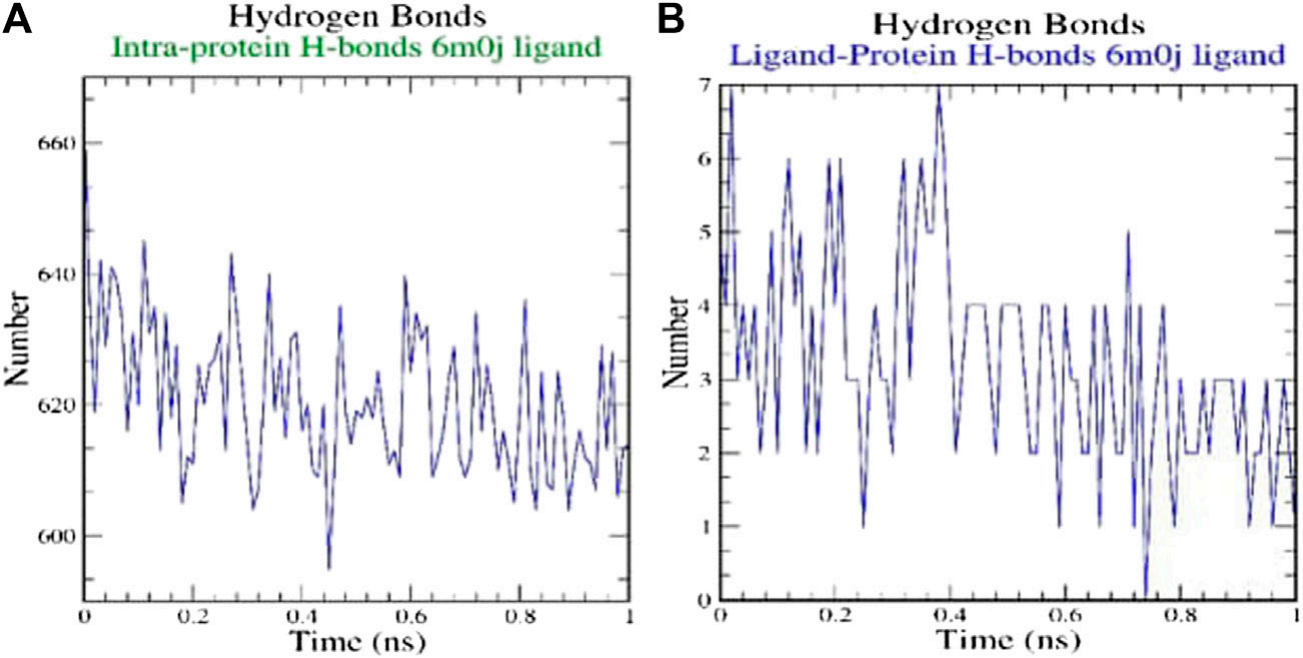
Analysis of hydrogen bonds on **(A)** 6M0J alone and **(B)** complex with the ligand over 10 ns simulation run.

H-bonds which appear during the Molecular Docking study being analyzed over the total simulation period. All the intermolecular H-bonds among ligands and protein were only considered during the analysis and plotted accordingly (Figure 3). It is evident from the plot that the number of H-bonds during the simulation runs remains consistent with Molecular Docking study, while few bonds were simultaneously broken and rebuilt.

**FIGURE 4:**
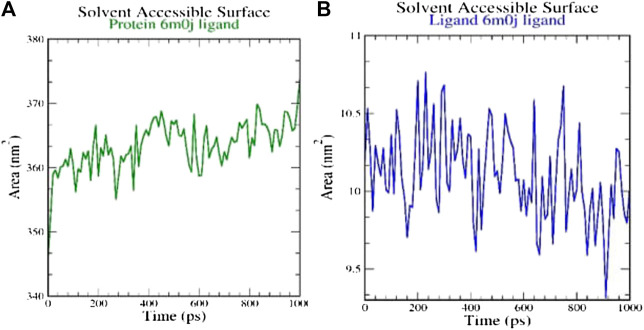
Analysis of solvent accessible surface area on **(A)** 6M0J alone and **(B)** complex with the ligand over 10 ns simulation run.

SASA measures the area around hydrophobic core formed between protein-ligand complexes (Figure 4). Consistent SASA values were observed.

## 4 Conclusion

To sum up, for the A. muricata phytochemicals with known potent antioxidant and anti‐inflammatory properties, in silico studies suggest that the Annonaceous Acetogenins show a good inhibition activity against the SARS‐CoV‐2 spike protein as per the Molecular Docking and MD simulation results obtained. Notwithstanding, *cis*-Annonacin was identified to be the most potent acetogenin with a low binding energy (indicative of the highest binding affinity) and greater hydrogen bond formation potential. This information could be useful in complementing the experimental data as the starting point for the development of new therapeutic drugs based on these molecules.

## Data Availability Statement

The original contributions presented in the study are included in the article/Supplementary Material, further inquiries can be directed to the corresponding authors.

## Author Contributions

SKP Research; SP: Research; CS: Research; SPK: Research and Writing the Manuscript; AS: Research; NM: Research; CE: Research; M‐AG: Research; OK: Research; WC: Research; KCP‐I: Research; JOC: Research; JF: Research; NF‐H: Research; DG‐M: Research and Writing the Manuscript.

## Funding

Researchers Supporting Project number (RSP‐2020/201), King Saud University, Riyadh, Saudi Arabia.

## Conflict of Interest

The authors declare that the research was conducted in the absence of any commercial or financial relationships that could be construed as a potential conflict of interest.
