# Supplementary material for: Evaluation of Annona muricata Acetogenins as Potential Anti-SARS-CoV-2 Agents Through Computational Approaches
Source: Front Chem. 2021 Jan 27;8:624716. doi: 10.3389/fchem.2020.624716 (PMC7958878; doi:10.3389/fchem.2020.624716)
Supplement: Supplementary file 1 [file datasheet1.pdf]

Supplementary Data

Evaluation of *Annona muricata* Acetogenins as Potential Anti-SARS-Cov-2 Agents Through Computational Approaches

SARS-CoV-2 Spike Protein – Crystal structure

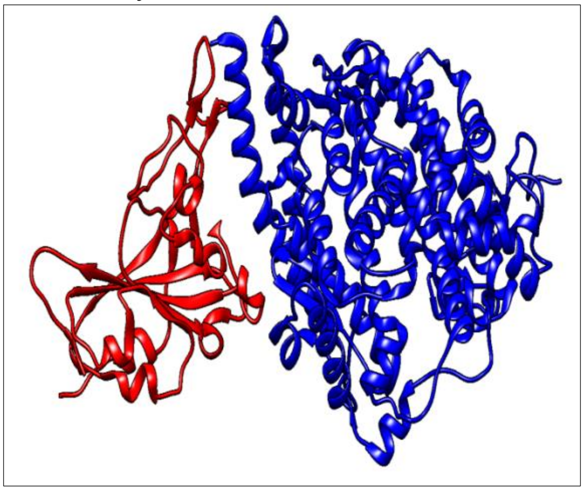

Figure S1: The crystal structure of corona virus spike (S) receptor (blue colored) - binding domain bound with ACE2 receptor (red colored) (PDB ID: 6M0J).

Ligand-Protein Interaction

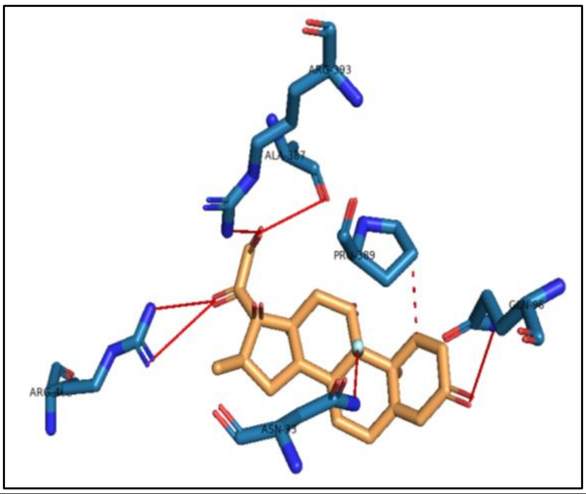

Hydrophobic Interactions

Index	Residue	AA	Distance	Ligand Atom	Protein Atom
1	389A	PRO	3.64	17	3033

Hydrogen Bonds

Index	Residue	AA	Distance H-A	Distance D-A	Donor Angle	Protein donor?	Sidechain	Donor Atom	Acceptor Atom
1	96A	GLN	2.38	3.24	145.33	✓	✓	670 [Nam]	23 [O2]
2	387A	ALA	2.45	3.33	142.86	✗	✗	26 [O3]	3017 [O2]
3	393A	ARG	1.89	2.85	162.58	✓	✓	3072 [Ng+]	26 [O3]
4	403E	ARG	2.36	3.31	160.96	✓	✓	5449 [Ng+]	25 [O2]
5	403E	ARG	2.71	3.57	145.33	✓	✓	5448 [Ng+]	25 [O2]

Halogen Bonds

Index	Residue	AA	Distance	Donor Angle	Acceptor Angle	Donor Atom	Acceptor Atom
1	33A	ASN	3.05	135.51	123.49	28 [F]	154 [Nam]

Figure S2: The detailed interaction of dexamethasone against SARS-CoV-2 spike protein.

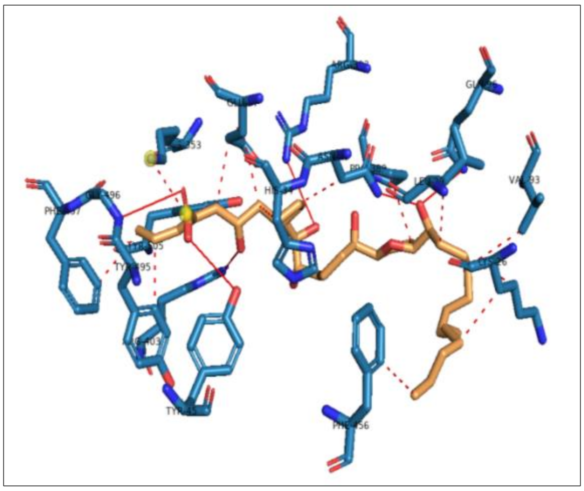

Hydrophobic Interactions

Index	Residue	AA	Distance	Ligand Atom	Protein Atom
1	26A	LYS	3.42	17	107
2	26A	LYS	3.46	10	108
3	29A	LEU	3.87	9	134
4	33A	ASN	3.47	26	170
5	34A	HIS	3.99	28	178
6	37A	GLU	3.64	30	203
7	37A	GLU	3.64	28	202
8	93A	VAL	3.76	11	663
9	389A	PRO	3.83	6	3050
10	456E	PHE	3.68	20	5885
11	495E	TYR	3.54	34	6188
12	497E	PHE	3.64	35	6206
13	505E	TYR	3.84	30	6264

Hydrogen Bonds

Index	Residue	AA	Distance H-A	Distance D-A	Donor Angle	Protein donor?	Sidechain	Donor Atom	Acceptor Atom
1	33A	ASN	1.93	2.80	145.63	✓	✓	172 [Nam]	37 [O3]
2	96A	GLN	3.30	3.84	116.56	✓	✓	688 [Nam]	37 [O3]
3	393A	ARG	3.31	4.03	131.57	✓	✓	3090 [Ng+]	42 [O3]
4	403E	ARG	2.37	2.88	112.00	✓	✓	5466 [Ng+]	41 [O3]
5	453E	TYR	2.91	3.80	159.87	✓	✓	5859 [O3]	40 [O.co2]
6	453E	TYR	2.89	3.80	157.23	✗	✓	40 [O.co2]	5859 [O3]
7	496E	GLY	3.25	3.92	126.20	✓	✗	6194 [Nam]	39 [O.co2]

Figure S3: The detailed interaction of Annomuricin A against SARS-CoV-2 spike protein.

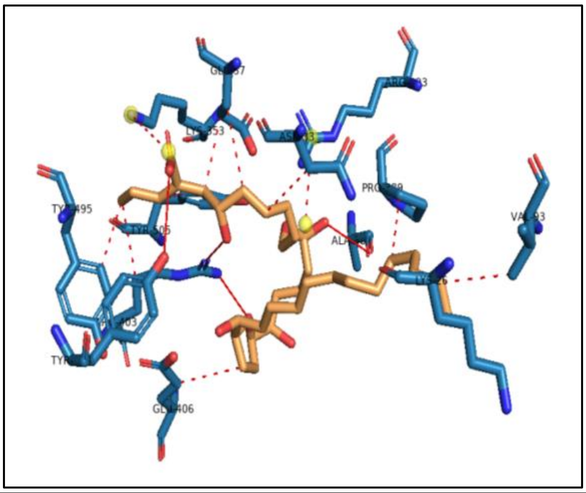

Hydrophobic Interactions ----

Index	Residue	AA	Distance	Ligand Atom	Protein Atom
1	26A	LYS	3.63	19	106
2	33A	ASN	3.97	26	168
3	37A	GLU	3.65	29	201
4	37A	GLU	3.97	27	200
5	93A	VAL	3.62	18	661
6	389A	PRO	3.81	15	3048
7	403E	ARG	3.96	34	5460
8	406E	GLU	3.83	5	5483
9	495E	TYR	3.75	34	6186
10	505E	TYR	3.96	31	6260
11	505E	TYR	3.59	29	6262

Hydrogen Bonds ——

Index	Residue	AA	Distance H-A	Distance D-A	Donor Angle	Protein donor?	Sidechain	Donor Atom	Acceptor Atom
1	387A	ALA	2.22	3.11	151.77	✖	✖	42 [O3]	3033 [O2]
2	403E	ARG	2.39	2.91	112.15	✔	✔	5464 [Ng+]	41 [O3]
3	403E	ARG	2.68	3.06	103.73	✔	✔	5465 [Ng+]	35 [O3]
4	453E	TYR	3.14	4.01	154.74	✔	✔	5857 [O3]	40 [O.co2]

Figure S4: The detailed interaction of Annomuricin B against SARS-CoV-2 spike protein.

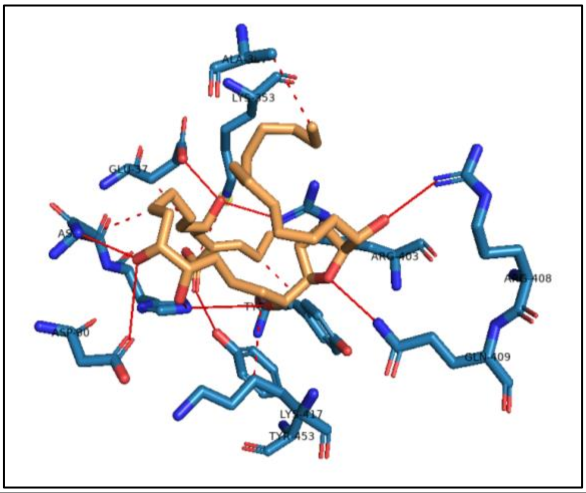

Hydrophobic Interactions - - - -

Index	Residue	AA	Distance	Ligand Atom	Protein Atom
1	33A	ASN	3.56	25	170
2	37A	GLU	3.60	30	202
3	387A	ALA	3.79	20	3036
4	417E	LYS	3.88	2	5565
5	495E	TYR	3.40	34	6188

Hydrogen Bonds ——

Index	Residue	AA	Distance H-A	Distance D-A	Donor Angle	Protein donor?	Sidechain	Donor Atom	Acceptor Atom
1	30A	ASP	2.99	3.55	117.89	✗	✓	42 [O3]	144 [O.co2]
2	33A	ASN	2.04	2.95	153.17	✓	✓	172 [Nam]	42 [O3]
3	34A	HIS	3.44	3.96	115.37	✗	✓	38 [O3]	183 [N2]
4	34A	HIS	3.02	3.97	162.71	✗	✓	43 [O3]	183 [N2]
5	37A	GLU	2.27	2.78	111.74	✗	✓	41 [O3]	206 [O.co2]
6	403E	ARG	2.47	3.09	120.88	✓	✓	5466 [Ng+]	41 [O3]
7	408E	ARG	2.12	2.80	123.90	✓	✓	5505 [Ng+]	37 [O3]
8	409E	GLN	2.52	2.99	108.85	✓	✓	5514 [Nam]	36 [O3]
9	453E	TYR	1.98	2.87	158.58	✓	✓	5859 [O3]	39 [O.co2]

Figure S5: The detailed interaction of Annomuricin C against SARS-CoV-2 spike protein.

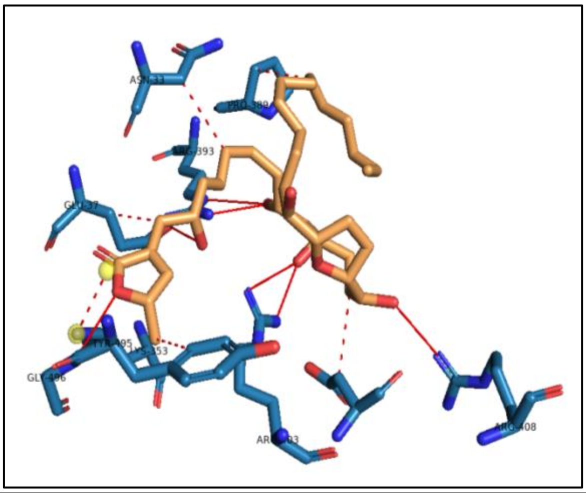

Hydrophobic Interactions ----

Index	Residue	AA	Distance	Ligand Atom	Protein Atom
1	33A	ASN	3.75	26	169
2	37A	GLU	3.50	30	201
3	389A	PRO	3.76	14	3049
4	405E	ASP	3.75	1	5475
5	495E	TYR	3.68	34	6187

Hydrogen Bonds —

Index	Residue	AA	Distance H-A	Distance D-A	Donor Angle	Protein donor?	Sidechain	Donor Atom	Acceptor Atom
1	37A	GLU	2.39	2.97	118.18	✖	✔	41 [O3]	205 [O.co2]
2	393A	ARG	1.94	2.92	169.79	✔	✔	3089 [Ng+]	42 [O3]
3	393A	ARG	3.20	3.92	132.55	✖	✔	42 [O3]	3086 [Ng+]
4	403E	ARG	2.04	2.96	155.37	✔	✔	5466 [Ng+]	43 [O3]
5	403E	ARG	3.18	3.84	125.61	✔	✔	5465 [Ng+]	43 [O3]
6	408E	ARG	2.73	3.21	110.63	✔	✔	5504 [Ng+]	38 [O3]
7	496E	GLY	2.85	3.71	146.52	✔	✖	6193 [Nam]	39 [O3]

Supplementary Figure 6: The detailed interaction of Muricatocin C against SARS-CoV-2 spike protein.

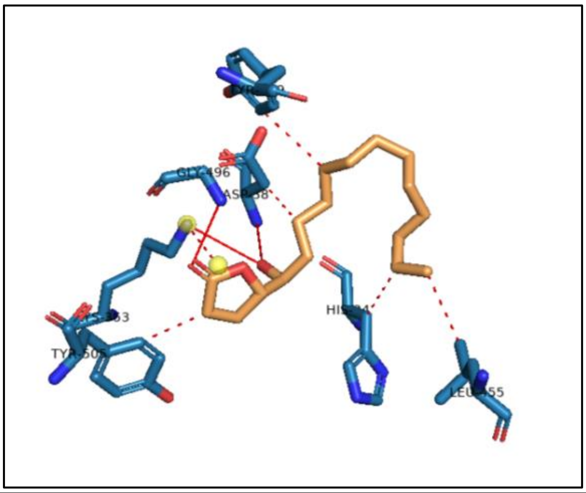

Hydrophobic Interactions ....

Index	Residue	AA	Distance	Ligand Atom	Protein Atom
1	34A	HIS	3.71	16	150
2	38A	ASP	3.42	1	183
3	449E	TYR	3.89	9	5788
4	455E	LEU	3.81	17	5850
5	505E	TYR	3.54	6	6234

Hydrogen Bonds —

Index	Residue	AA	Distance H-A	Distance D-A	Donor Angle	Protein donor?	Sidechain	Donor Atom	Acceptor Atom
1	38A	ASP	3.39	3.70	100.03	✔	✘	179 [Nam]	20 [O3]
2	353A	LYS	3.23	3.68	108.42	✔	✔	2736 [N3+]	20 [O3]
3	496E	GLY	1.99	2.86	147.15	✔	✘	6166 [Nam]	19 [O2]

Figure S7: The detailed interaction of Muricatacin against SARS-CoV-2 spike protein.

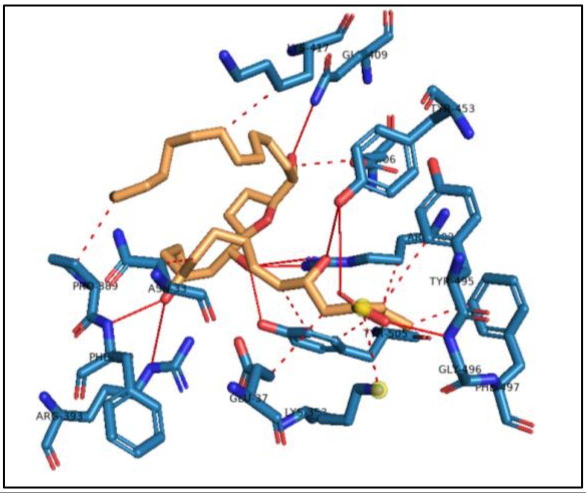

Hydrophobic Interactions ----

Index	Residue	AA	Distance	Ligand Atom	Protein Atom
1	33A	ASN	3.64	24	168
2	37A	GLU	3.87	30	201
3	389A	PRO	3.68	18	3048
4	403E	ARG	3.90	33	5460
5	406E	GLU	3.64	8	5483
6	417E	LYS	3.61	13	5563
7	495E	TYR	3.90	32	6186
8	497E	PHE	3.63	34	6204
9	505E	TYR	3.68	33	6260
10	505E	TYR	3.89	28	6262

Hydrogen Bonds ——

Index	Residue	AA	Distance H-A	Distance D-A	Donor Angle	Protein donor?	Sidechain	Donor Atom	Acceptor Atom
1	390A	PHE	2.53	3.01	109.66	✓	✗	3051 [Nam]	42 [O3]
2	393A	ARG	3.22	4.07	145.99	✗	✓	42 [O3]	3085 [Ng+]
3	403E	ARG	2.37	3.22	143.29	✓	✓	5464 [Ng+]	38 [O3]
4	403E	ARG	2.11	3.02	154.52	✓	✓	5465 [Ng+]	38 [O3]
5	409E	GLN	2.34	3.18	142.71	✓	✓	5512 [Nam]	37 [O3]
6	453E	TYR	3.11	4.01	162.10	✓	✓	5857 [O3]	39 [O.co2]
7	453E	TYR	3.31	3.75	109.43	✗	✓	41 [O3]	5857 [O3]
8	496E	GLY	2.77	3.19	106.41	✓	✗	6192 [Nam]	40 [O.co2]
9	505E	TYR	2.19	2.80	119.11	✗	✓	38 [O3]	6264 [O3]

Figure S8: The detailed interaction of cis-Annonacin against SARS-CoV-2 spike protein.

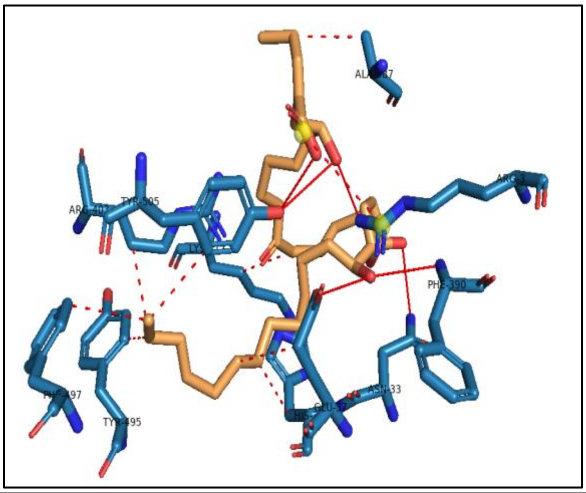

Hydrophobic Interactions ----

Index	Residue	AA	Distance	Ligand Atom	Protein Atom
1	34A	HIS	3.93	12	175
2	37A	GLU	3.68	13	200
3	387A	ALA	3.57	33	3033
4	403E	ARG	3.86	17	5459
5	417E	LYS	3.93	22	5562
6	495E	TYR	3.44	17	6185
7	497E	PHE	3.92	18	6203
8	505E	TYR	3.67	18	6259

Hydrogen Bonds —

Index	Residue	AA	Distance H-A	Distance D-A	Donor Angle	Protein donor?	Sidechain	Donor Atom	Acceptor Atom
1	33A	ASN	3.40	3.85	109.71	✔	✔	169 [Nam]	42 [O3]
2	37A	GLU	2.39	3.35	166.43	✖	✔	37 [O3]	203 [O.co2]
3	390A	PHE	2.76	3.30	115.12	✔	✖	3050 [Nam]	37 [O3]
4	393A	ARG	2.24	3.08	142.53	✔	✔	3087 [Ng+]	41 [O3]
5	505E	TYR	2.08	2.91	146.89	✔	✔	6263 [O3]	40 [O.co2]
6	505E	TYR	3.24	3.79	117.69	✖	✔	41 [O3]	6263 [O3]

Figure S9: The detailed interaction of Annonacin-10-one against SARS-CoV-2 spike protein.

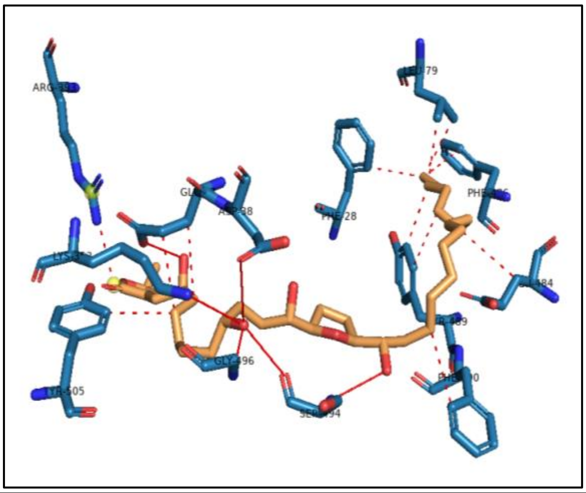

Hydrophobic Interactions ----

Index	Residue	AA	Distance	Ligand Atom	Protein Atom
1	28A	PHE	3.37	20	122
2	37A	GLU	3.90	28	199
3	37A	GLU	3.86	27	200
4	79A	LEU	3.43	18	545
5	79A	LEU	3.50	20	546
6	484E	GLU	3.96	15	6101
7	486E	PHE	3.75	20	6116
8	489E	TYR	3.83	16	6141
9	489E	TYR	3.45	18	6143
10	490E	PHE	3.94	8	6154
11	505E	TYR	3.66	27	6261

Hydrogen Bonds —

Index	Residue	AA	Distance H-A	Distance D-A	Donor Angle	Protein donor?	Sidechain	Donor Atom	Acceptor Atom
1	37A	GLU	1.89	2.70	137.88	✖	✔	41 [O3]	203 [O.co2]
2	38A	ASP	3.56	3.85	100.51	✔	✔	210 [O3]	42 [O3]
3	353A	LYS	2.54	3.38	138.40	✔	✔	2761 [N3+]	42 [O3]
4	494E	SER	2.52	3.15	120.11	✔	✖	6173 [N3]	37 [O3]
5	494E	SER	2.12	3.07	163.62	✖	✖	42 [O3]	6176 [O2]
6	496E	GLY	2.54	2.96	105.28	✔	✖	6191 [Nam]	42 [O3]

Figure S10: The detailed interaction of cis-Goniothalamicin against SARS-CoV-2 spike protein.

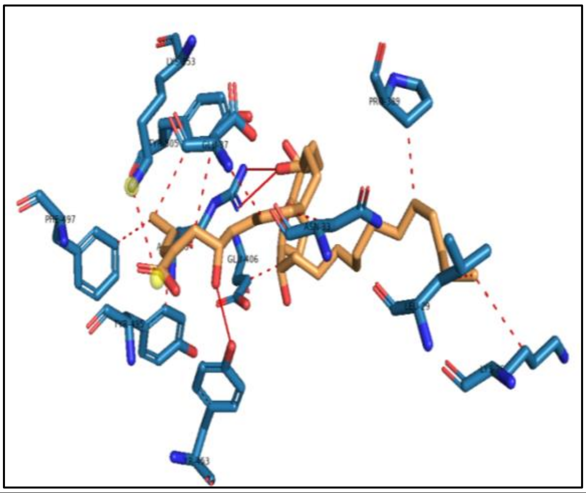

Hydrophobic Interactions

Index	Residue	AA	Distance	Ligand Atom	Protein Atom
1	26A	LYS	3.93	19	106
2	29A	LEU	3.76	19	132
3	33A	ASN	3.56	25	168
4	37A	GLU	3.86	30	201
5	37A	GLU	3.84	26	200
6	389A	PRO	3.57	15	3048
7	403E	ARG	3.73	34	5460
8	406E	GLU	3.93	5	5483
9	495E	TYR	3.81	33	6186
10	497E	PHE	4.00	34	6204
11	505E	TYR	3.79	34	6260

Hydrogen Bonds

Index	Residue	AA	Distance H-A	Distance D-A	Donor Angle	Protein donor?	Sidechain	Donor Atom	Acceptor Atom
1	403E	ARG	2.31	3.15	142.22	✔	✔	5465 [Ng+]	42 [O3]
2	403E	ARG	2.04	2.95	153.97	✔	✔	5464 [Ng+]	42 [O3]
3	453E	TYR	2.06	2.85	141.57	✔	✔	5857 [O3]	41 [O3]

Figure S11: The detailed interaction of Arianacin against SARS-CoV-2 spike protein.

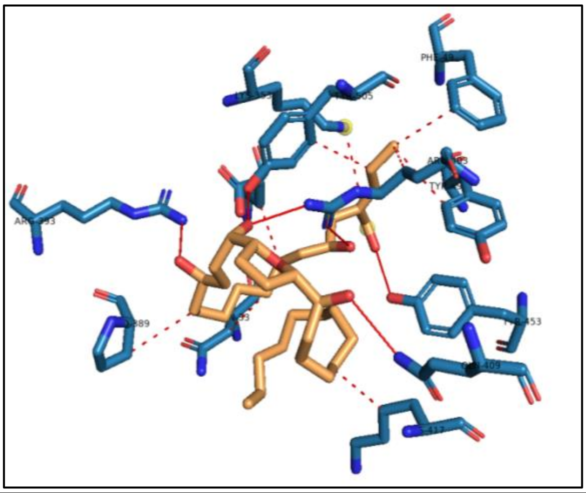

Hydrophobic Interactions ----

Index	Residue	AA	Distance	Ligand Atom	Protein Atom
1	33A	ASN	3.63	26	168
2	37A	GLU	3.91	27	200
3	389A	PRO	3.96	23	3048
4	403E	ARG	3.88	34	5460
5	417E	LYS	3.73	11	5563
6	495E	TYR	3.73	34	6186
7	497E	PHE	3.68	34	6204
8	505E	TYR	3.78	33	6260

Hydrogen Bonds —

Index	Residue	AA	Distance H-A	Distance D-A	Donor Angle	Protein donor?	Sidechain	Donor Atom	Acceptor Atom
1	393A	ARG	2.04	3.00	166.55	✔	✔	3088 [Ng+]	42 [O3]
2	403E	ARG	2.07	2.91	141.92	✔	✔	5464 [Ng+]	41 [O3]
3	403E	ARG	1.93	2.87	158.74	✔	✔	5465 [Ng+]	38 [O3]
4	409E	GLN	2.38	3.23	145.13	✔	✔	5512 [Nam]	37 [O3]
5	453E	TYR	2.10	3.01	163.61	✔	✔	5857 [O3]	40 [O.co2]
6	453E	TYR	2.63	3.01	103.93	✖	✔	40 [O.co2]	5857 [O3]
7	505E	TYR	2.48	2.87	105.02	✔	✔	6264 [O3]	38 [O3]

Figure S12: The detailed interaction of Javoricin against SARS-CoV-2 spike protein.

Optimized Molecular Structures:

Before Docking

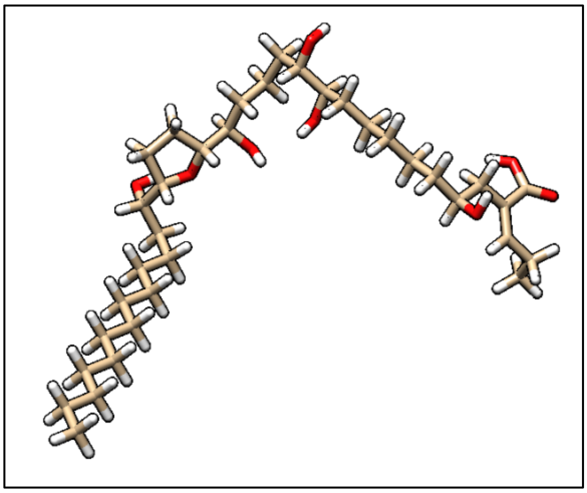

(a)

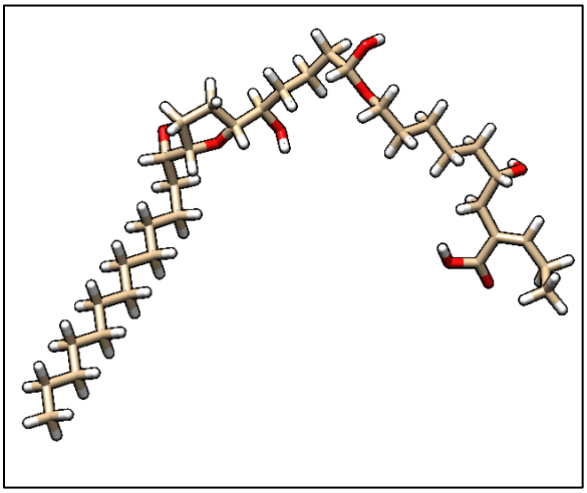

(b)

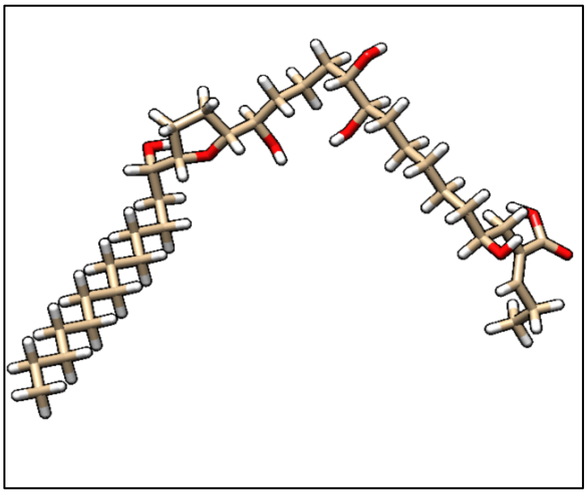

(c)

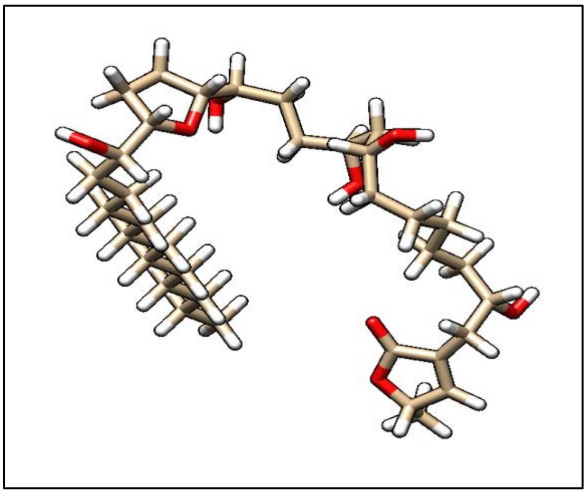

(d)

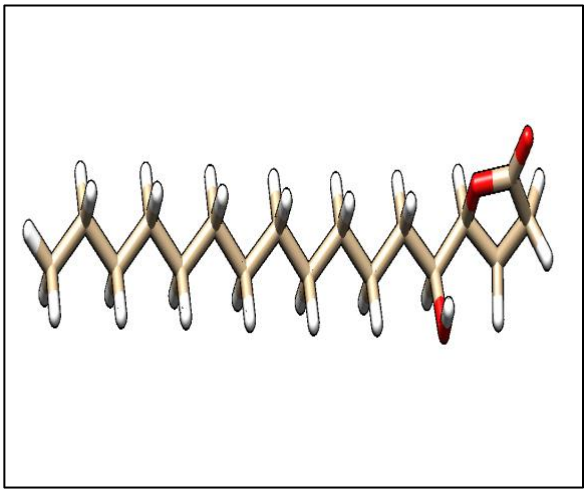

(e)

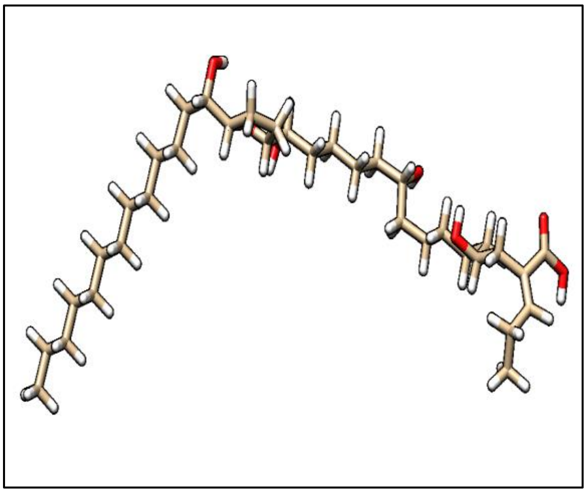

(f)

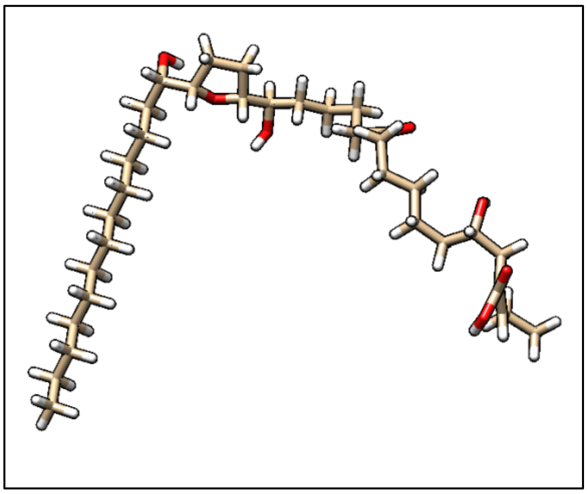

(g)

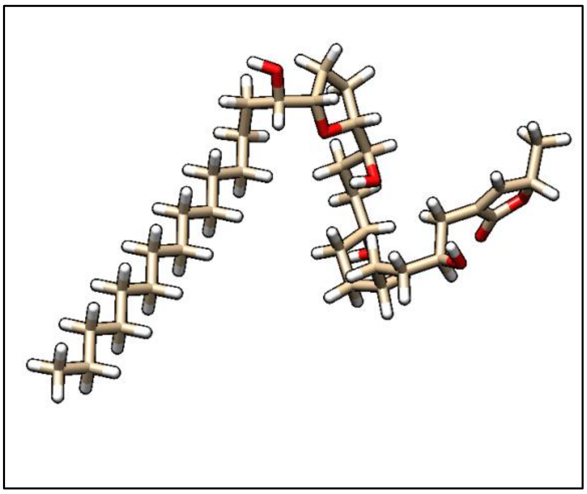

(h)

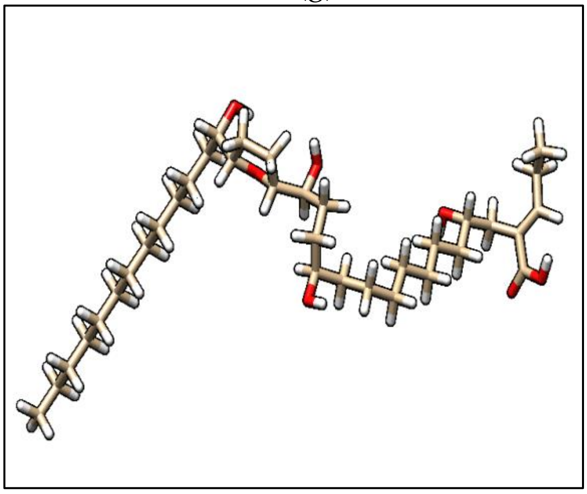

(i)

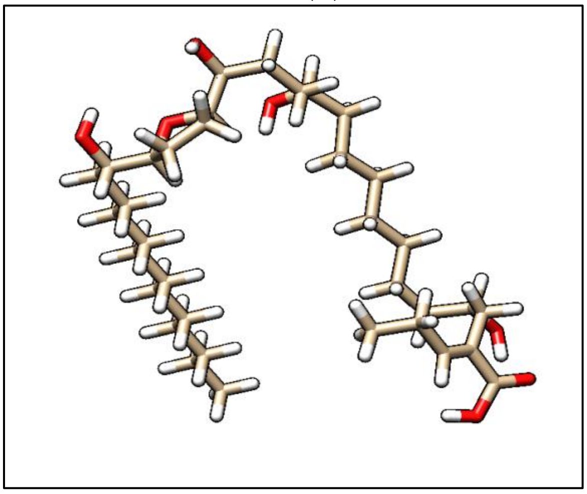

(j)

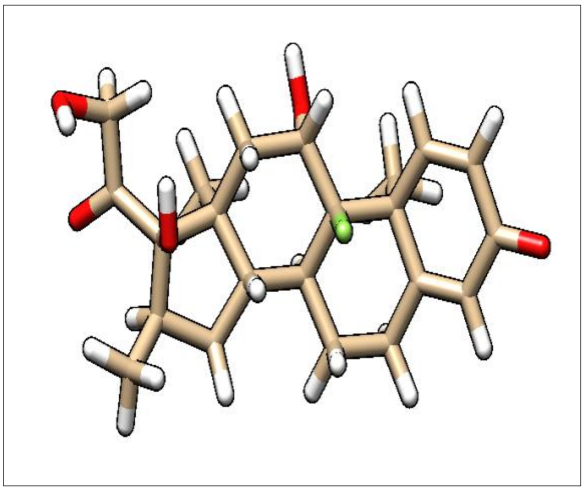

(k)

Figure S13: Optimized molecular structures of (a) Annomuricin A, (b) Annomuricin B, (c) Annomuricin C, (d) Muricatocin C, (e) Muricatacin, (f) cis-Annonacin, (g) Annonacin-10-one, (h) cis-Goniothalamicin, (i) Arianacin, (j) Javoricin and (k) Dexamethasone, before docking to the spike protein.

After Docking

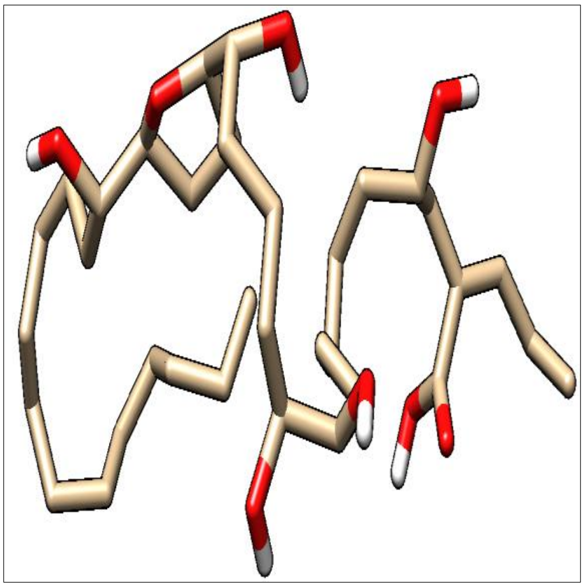

(a)

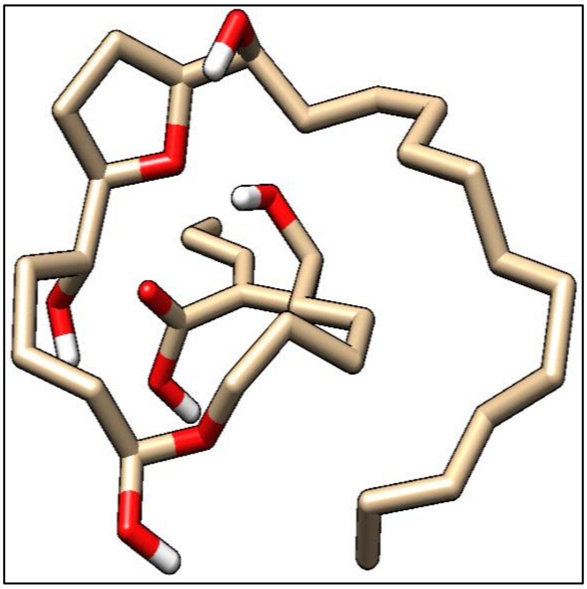

(b)

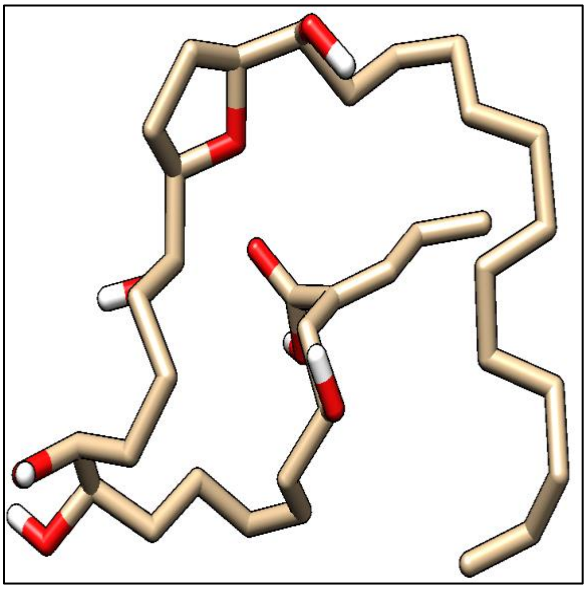 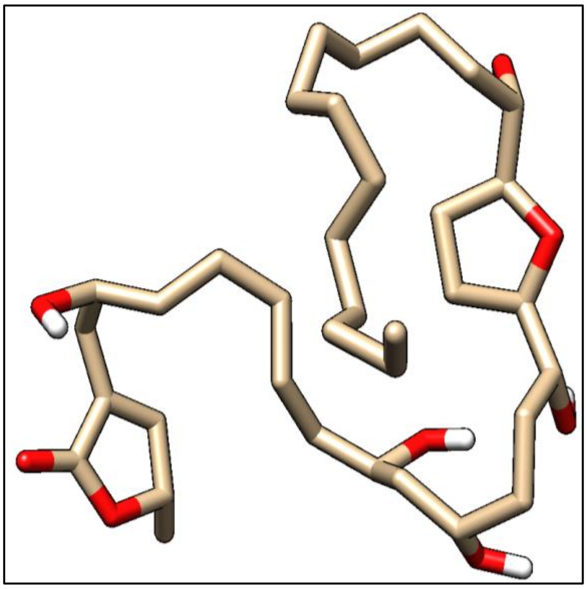

(c) (d)

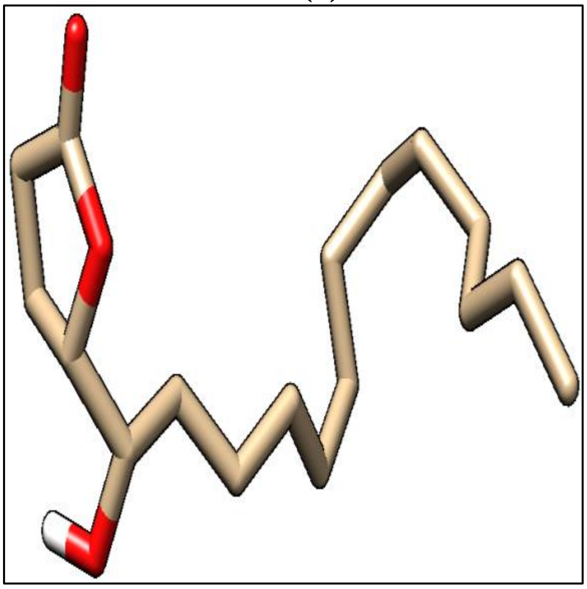 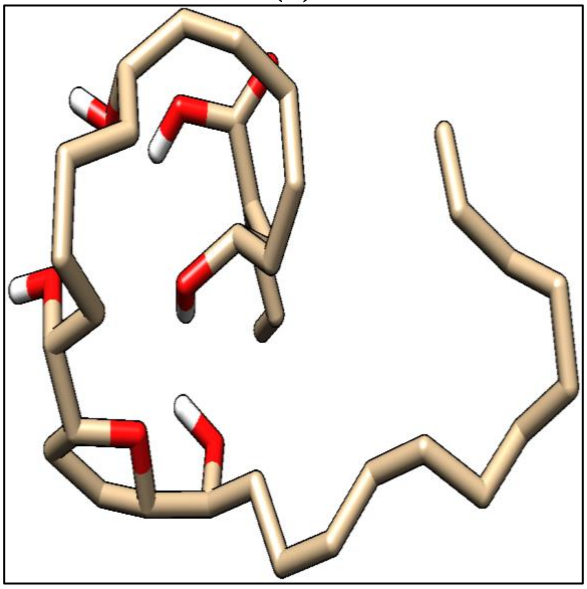

(e) (f)

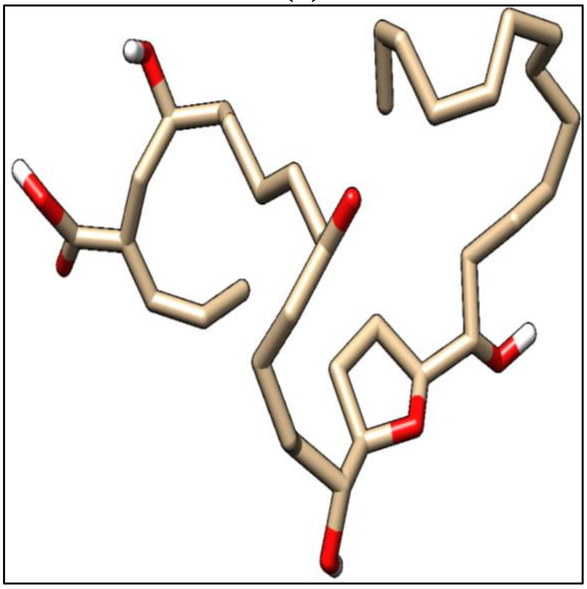 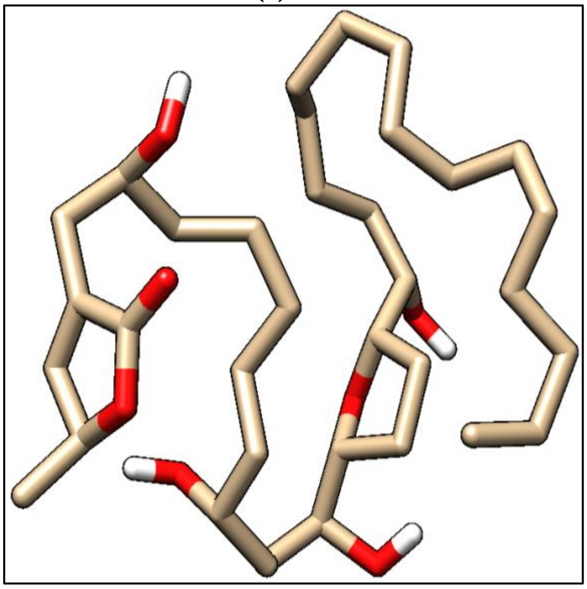

(g) (h)

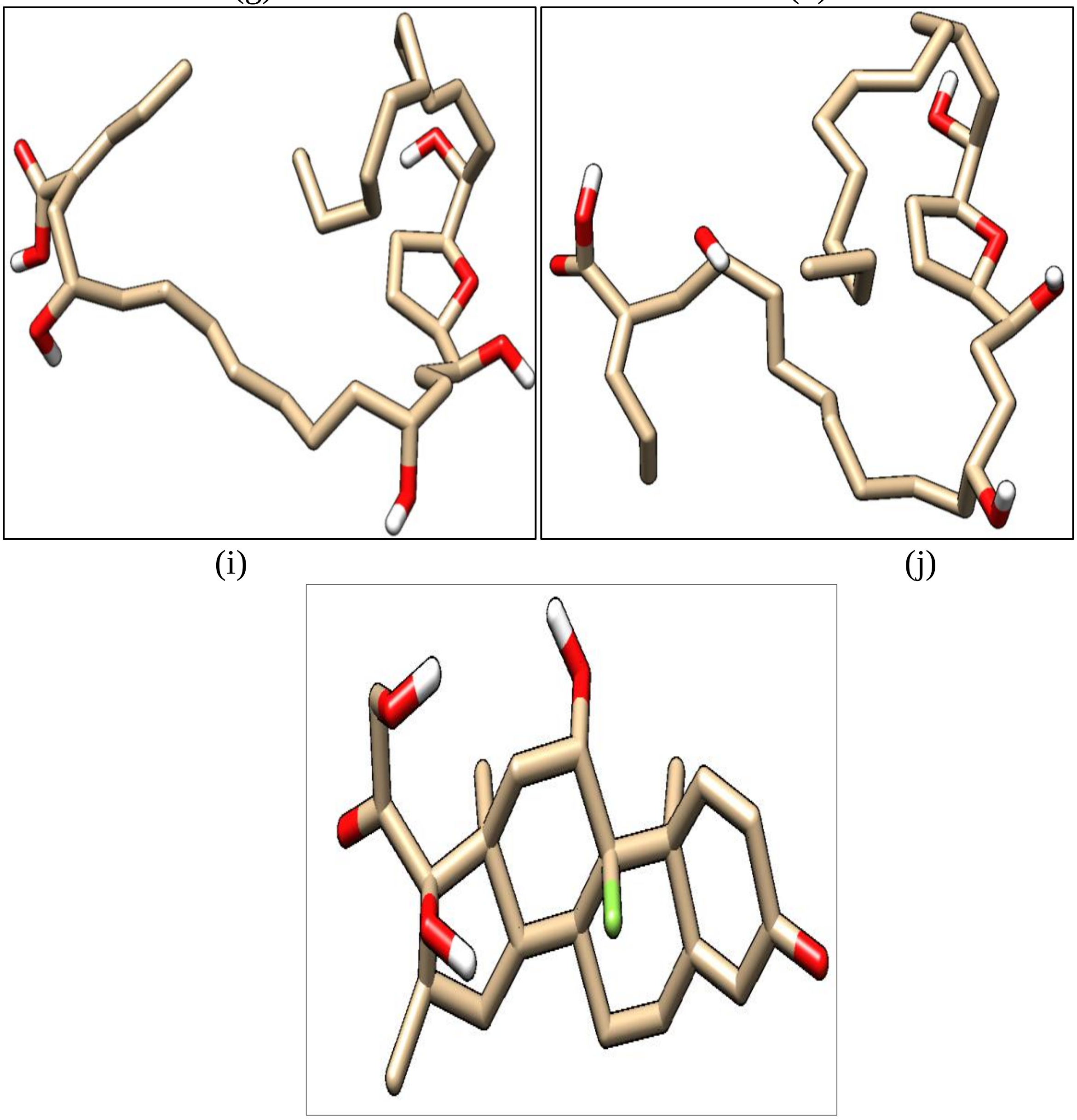

(k)

==Figure S14:== 3-dimensional pictures of the ligands obtained after the Molecular docking interaction studies (a) Annomuricin A, (b) Annomuricin B, (c) Annomuricin C, (d) Muricatocin C, (e) Muricatacin, (f) cis-Annonacin, (g) Annonacin-10-one, (h) cis-Goniothalamicin, (i) Arianacin, (j) Javoricin and (k) Dexamethasone.
